# FOXM1 functions collaboratively with PLAU to promote gastric cancer progression

**DOI:** 10.7150/jca.37323

**Published:** 2020-01-01

**Authors:** Chao Ai, Jixin Zhang, Shenyi Lian, Jie Ma, Balázs Győrffy, Zhenyuan Qian, Yong Han, Qin Feng

**Affiliations:** 1 Department of Pharmacy, Beijing Tsinghua Changgung Hospital, School of Clinical Medicine, Tsinghua University, Beijing 102218, P. R. China.; 2 Department of Pathology, Peking University First Hospital, Beijing, China; 3 Key laboratory of Carcinogenesis and Translational Research (Ministry of Education), Department of Pathology, Peking University Cancer Hospital &Institute, Beijing, China; 4 Department of Pathology, Zhejiang Provincial People's Hospital, Hangzhou, Zhejiang, China; 5 Momentum Cancer Biomarker Research Group, Institute of Enzymology, Hungarian Academy of Sciences, Budapest, H-1117, Hungary; Second Department of Pediatrics, Semmelweis University, Budapest, H-1094, Hungary; 6 Department of Gastrointestinal Surgery, Zhejiang Provincial People's Hospital, Hangzhou, Zhejiang, China; 7 Clinical Research Institute, Zhejiang Provincial People's Hospital, Hangzhou, Zhejiang, China

**Keywords:** FOXM1, PLAU, gastric cancer, immune infiltration, chemo-resistance

## Abstract

**Background:** Gastric cancer (GC) is one of the main mortality cause worldwide. Previously, we found Forkhead box protein (FOXM1) or Urokinase-type plasminogen activator (PLAU) are independent prognostic markers of GC. This study aims to explore the combining prognostic efficacy and the potential insights underlying additive effect of FOXM1 to PLAU in GC progression through in-silico analyses.

**Method:** The expression of FOXM1 and PLAU were profiled in 33 cancer types using public data. A merged GC expression dataset containing 598 samples was used for evaluating prognostic significance of FOXM1/PLAU. Gene Set Enrichment Analysis (GSEA) was performed to elucidate the mechanisms underlying FOXM1/PLAU promoted GC progression. The Cancer Genome Atlas (TCGA) was used for analyzing the association between FOXM1/PLAU and tumor immune infiltration. Genomic and proteomic differences between FOXM1+PLAU+ and FOXM1-PLAU- groups were also computed using TCGA GC data. Drugs targeting FOXM1/PLAU associated gene expression pattern was analyzed using LINCs database.

**Results:** FOXM1 and PLAU are overexpressed in 17/33 cancer types including GC. Kaplan-Meier analyses indicate that the FOXM1+PLAU+ subgroup have the worst prognosis, while FOXM1-PLAU- subgroup have the best survival. Bioinformatics analysis indicated that FOXM1+PLAU+ associated genes are enriched in TGF-beta, DNA repair and drug resistance signaling pathways; FOXM1 and PLAU expression are negatively correlated with tumor immune infiltration. Genomic and proteomic differences between FOXM1+PLAU+ and FOXM1-PLAU- groups were presented. Data mining from LINCs suggested several chemicals or drugs that could target the gene expression pattern of FOXM1+PLAU+ patients.

**Conclusion:** FOXM1+PLAU+ can serve as effective prognostic biomarkers and potential therapeutic targets for GC. Due to the additive effect of these two genes, screening for drugs or chemicals that targeting the expression patterns PLAU+FOXM1+ subgroup may exert important clinical impact on GC management.

## Introduction

Although the incidence of gastric cancer decreased dramatically in recent years, it remains one of the main cause of mortality worldwide^1^. To some extent, the screening and therapy for the early stage gastric cancer patients is mature and effective, however, the survival of the late stage patients with distal metastasis is still very poor^2^. FOXM1, also named box protein M1, is known as a member of Forkhead box (Fox) superfamily of transcription factor that regulate the cell cycle, DNA synthesis and cell proliferation^3^-^5^. FOXM1 controls the transition of G1/S and G2/M phase by regulating the expression of AuroB kinase, CDC25B and P27^6^-^8^. FOXM1 triggers the DNA damage repair signaling by persistence activation of ATM-CHK2 signal axis and related to the MELK- mediates the oncogenic activation^9^, ^10^. FOXM1 is highly expressed in many MST (malignant solid tumors) such as non-small cell lung cancer^11^, colorectal cancer^12^, esophageal cancer^13^, ^14^, gallbladder cancer^15^, gastric cancer^16^, glioblastoma^17^, ^18^ and hepatocellular carcinoma^19^, ^20^. Most studies indicated the high expression of FOXM1 is related to the poor prognosis of patients of MST^21^, ^22^. FOXM1 plays an important role in the development of embryo, adult tissue homeostasis and tumorigenesis and metastasis, which including the process of cell senescence, cell migration and invasion^23^. Siomycin A, a FOXM1 inhibitor, downregulate the level of FOXM1in the metastatic melanoma cell lines and induce cell apoptosis^24^. Down regulation of FOXM1 expression the proliferation and migration of hepatocellular carcinoma and renal cancer^25^-^27^. It was also reported that FOXM1 could promote gastric cancer cell migration and invasion^28^. FOXM1 signaling is one of the key pathways in ovarian cancer development and targeting FOXM1 is an effective therapeutic strategy^29^, ^30^. Moreover, the overexpression of FOXM1 could induce the apoptosis and cell senescence, which was related to the ROS reaction and chemo-resistance^31^. Knockdown of the FoxM1 enhances the sensitivity of ovarian cancer and gastric cancer cells to cisplatin^32^, ^33^.

PLAU, also named Urokinase-type plasminogen activator (uPA). Unlike other Urokinases, PLAU does not possess the kinase activity; it performs as a protease, which belongs to the serine peptidase of S1 of Clan PA^34^. PLAU participate the transition of plasminogen to plasmin, and proteolyzes the proteins related to the ECM remodeling and activates growth factors^35^. PLAU activates the MAPK, Jak-Stat signal pathway and focal adhesion kinase systems by its direct binding to the uPAR (Urokinase- plasminogen activator receptor) or indirectly by activation of plasmin which releases growth factors from ECM^36^. The status of PLAU is also the biomarker of prognostic and predictive factor in breast cancer and non-small cell lung cancer^37^, ^38^ and synthetic antibody against PLAU has been demonstrated the inhibition ability to the cancer progression^39^. Using chicken embryo system, researchers demonstrated that PLAU play an important role in the early process of tumor cell dissemination which is the initial deliberation of cancer cell from the primary sites^40^. And in the mice PLAU or plasminogen deficient model, the distal metastasis such as lung and lymph node metastasis was decreased while no effect on the growth of tumor^41^, ^42^. Wang et al. showed that inhibition of PLAU expression could suppress the migratory and invasive ability of cervical cancer cells through down regulating MMP2 expression^43^.

Previously, we found that overexpression of FOXM1 and PLAU were associated with gastric cancer progression and poor prognosis^44^. In the current study, several bioinformatics approaches were employed to explore the molecular mechanisms underlying FOXM1+/PLAU+ related malignant phenotype and potential therapeutic options.

## Patients and methods

### Ethics Statement

All the gastric cancer gene expression profiling datasets were obtained from publicly available resources, The Research Ethics Committee of Zhejiang Provincial People's Hospital waived the requirement for ethical approval.

### Genomic Analysis

Gastric cancer gene expression profiling dataset for Kaplan-Meier analyses were set up using raw data obtained from GEO (http://www.ncbi.nlm.nih.gov/geo/)^45^. Briefly, keywords “gastric”, “cancer”, “GPL96”, and “GPL570” were utilized. Only publications with raw data, survival information and at least 30 patients within the dataset were included. Affymetrix HG-U133 Plus 2.0 (GPL570) and HG-U133A (GPL96) gene chips were chosen because their probe sets are overlapped. By utilizing the Affy Bioconductor library in R (http://www.r-project.org), raw CEL files were MAS5 normalized. Finally, to set the average expression on each chip to 1000 to reduce batch effects, a second scaling normalization was performed as described^46^, ^47^. This merged dataset was used in survival analyses. Computation of differentially expressed genes and two class Gene Set Enrichment Analysis (GSEA)^48^ were performed using data from Gene Expression Omnibus (GEO, accession number:GSE15459^49^). Gene-Drug interaction data was downloaded from LINCs dataset^50^. All other data are from The Cancer Genome Atlas (TCGA: http://cancergenome.nih.gov/) and GEO (accession no.GSE27342^51^). Differential expression analyses and immune infiltration analysis were using TIMER^52^. Gene network visualization was presented through GeneMAINA^53^.

### Statistical Analyses

Standard statistical tests including log rank test, fisher exact test and independent samples t-test were employed in the data analyses. Adjust P value was corrected for multiple comparisons using the Benjamini and Hochberg's false discovery rate^54^. Significance was defined as a P value < 0.05. GraphPad Prism 5.01 (GraphPad Software, Inc. [www.graphpad.com]) and R 3.2.1 (R Foundation for Statistical Computing [http://www.r-project.org/]) were utilized to perform all the analyses.

## Results

### Expression of FOXM1 and PLAU in 33 cancer types and association with genetic alteration

We analyzed FOXM1 and PLAU mRNA expression in different cancer types using TIMER^52^. The results indicated that FOXM1 and PLAU are overexpressed in 17 cancer types including GC (Supplementary Figure 1). Expression data from GEO (accession no.GSE27342) confirmed the overexpression of FOXM1 and PLAU in GC compare with gastric mucosa (p < 0.0001 and 0.0001, respectively. Supplementary Figure 2A). GC data from TCGA showed that FOXM1 and PLAU expression were negatively correlated with methylation levels (r = 0.26 and 0.16, p < 0.0001 and 0.0001, respectively. Supplementary Figure 2B). FOXM1 expression is positively correlated with copy number variation (CNV), while there are no correlation between PLAU expression and CNV (Supplementary Figure 2C). Taken tumor cell purity into consideration, FOXM1 and PLAU expression are positively correlated (r = 0.163, p = 0.00143. Supplementary Figure 2D). There were no statistical correlation between FOXM1 or PLAU expression and GC stages (Supplementary Figure 3).

**PLAU+FOXM1+ predicted worse outcome in gastric cancer**

**Figure 1:**
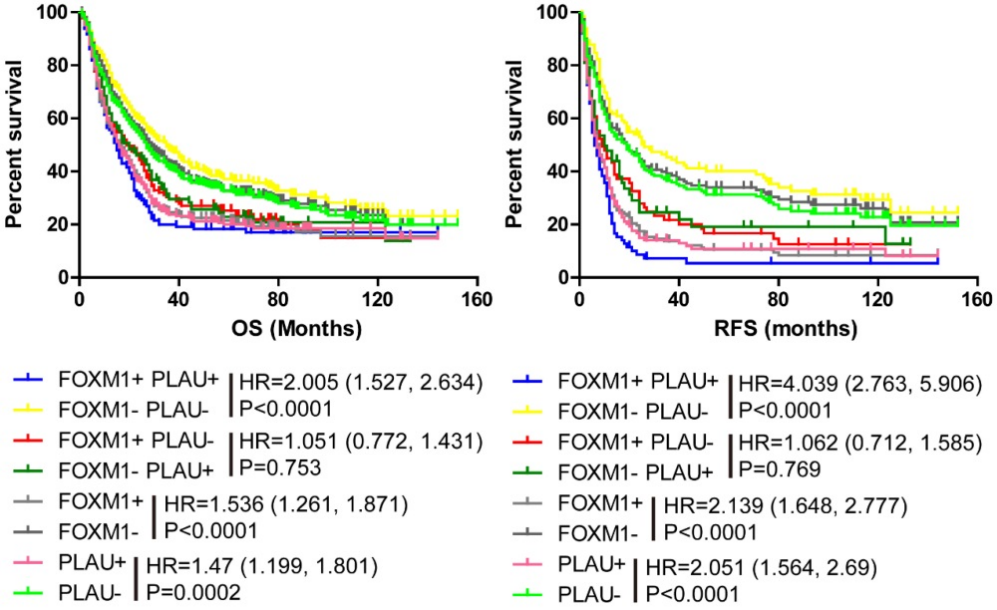
Kaplan-Meier survival analyses of gastric cancer patients stratified by FOXM1 and PLAU status. (Left) OS analysis of gastric cancer patients using pooled gastric cancer dataset (N=598). (Right) RFS analysis of gastric cancer patients using pooled gastric cancer dataset (N=363). In pooled gastric cancer dataset, FOXM1+ or PLAU+ were defined as ≥ median expression value of each gene, respectively. Different line colors represents different groups. The hazard ratio and log rank p value presented.

Kaplan-Meier analysis and log-rank test were performed using our merged Győrffy dataset and the results are in accordance with the previous results. The FOXM1 negative subgroup had longer OS and RFS than FOXM1positive subgroup (Figure 1, Left and Right, p<0.0001, HR (95%CI) =1.536(1.261-1.871), p<0.0001, HR (95%CI) =2.139(1.648-2.77)). Meanwhile, the PLAU negative subgroup patients had longer OS and RFS than PLAU positive subgroup (Figure 1, Left and Right, p<0.0001, HR (95%CI) =1.47(1.199-1.801); p<0.0001, HR (95%CI) =2.051(1.564-2.69)). When combine FOXM1 with PLAU to analysis OS and RFS, similar conclusion were obtained. The PLAU-FOXM1- subgroup got the best prognosis, while PLAU+ FOXM1+ subgroup had the worst prognosis (Figure 1, Left and Right, p<0.0001). The sample number of each group is presented in Supplementary table 1.

### Molecular patterns of PLAU+FOXM1+ subgroup compared with PLAU-FOXM1- subgroup

**Figure 2:**
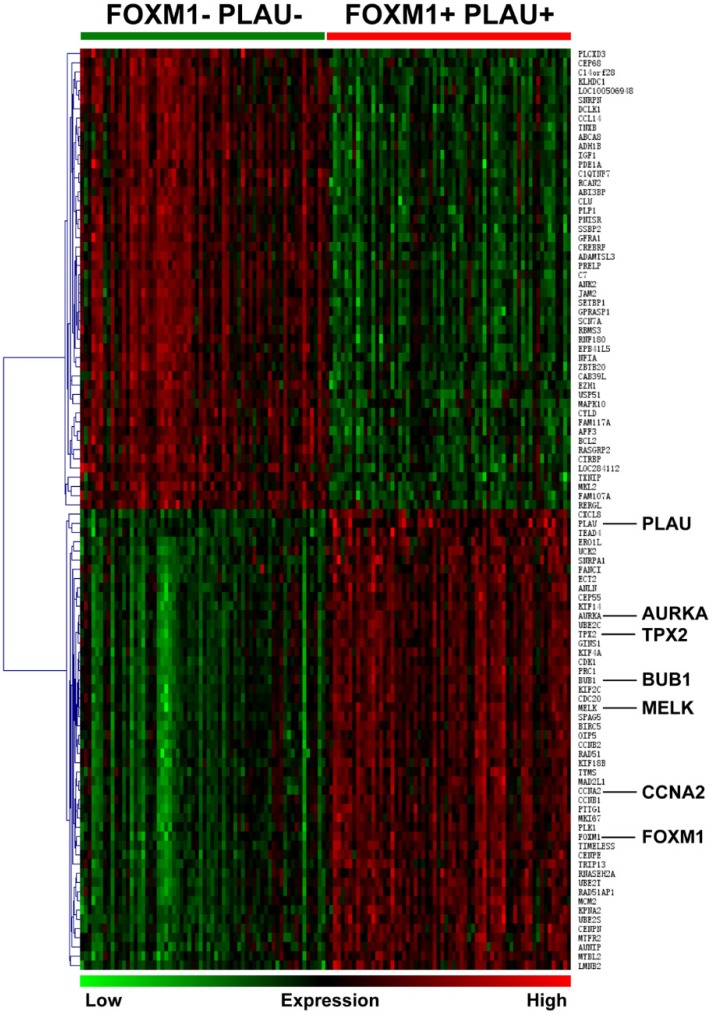
This heat map shows top 50 up-regulated genes and top 50 down-regulated genes in FOXM1+/PLAU+ subgroup compared with FOXM1-/PLAU- subgroup. From this graph, we could see that genes such as FOXM1, PLAU, AURKA, TPX2, BUB1, MELK and CCNA2 are overexpressed in FOXM1+/PLAU+ subgroup. Green grid represents low expression while red represents high expression.

Differentially expressed genes between PLAU-FOXM1- and PLAU+FOXM1+ subgroups were computed using publicly available dataset from GEO (GSE15459, subgroups were divided as follows. PLAU-FOXM1-: PLAU<median expression value and FOXM1<median expression value; PLAU+FOXM1+: PLAU>median expression value and FOXM1>median expression value). Top 50 up regulated and down regulated genes in PLAU+FOXM1+ group compared with PLAU-FOXM1- group were visualized in Figure 2 and gene details are listed in Supplementary Table 2. From this table, we could see that genes such as PRC1, SPAG5, AURKA, TPX2, BUB1, MELK and CCNA2 were up regulated in PLAU+FOXM1+ subgroup in comparison with PLAU-FOXM1- subgroup, which participate in diverse aspect of physiological and pathological process, such as NF-kB pathway, cytoskeletal signaling, adheren junctions remodeling, chromosome remodeling, cell cycle, calcium channel, DNA repair and TGF-beta signaling pathways.

By comparing the mutation and clinical attributes between FOXM1+PLAU+ and FOXM1-PLAU- groups using TCGA GC data, we show that the mutation count and fraction of genome altered in FOXM1+PLAU+ group are significantly higher than FOXM1-PLAU- group (Supplementary Figure 4 and Supplementary Table 3). JARID2, ZFHX4 and SYNE1 mutations are significantly enriched in FOXM1+PLAU+ group (Supplementary Figure 4 and Supplementary Table 4). Comparison of top genes' mutation frequency and CNA frequency between FOXM1+PLAU+ and FOXM1-PLAU- groups are presented in Supplementary Figure 5. Through analyzing RPPA data from TCGA GC dataset, the protein changes of key signaling pathways between FOXM1+PLAU+ and FOXM1-PLAU- groups were listed in Supplementary Table 5.

**Figure 3:**
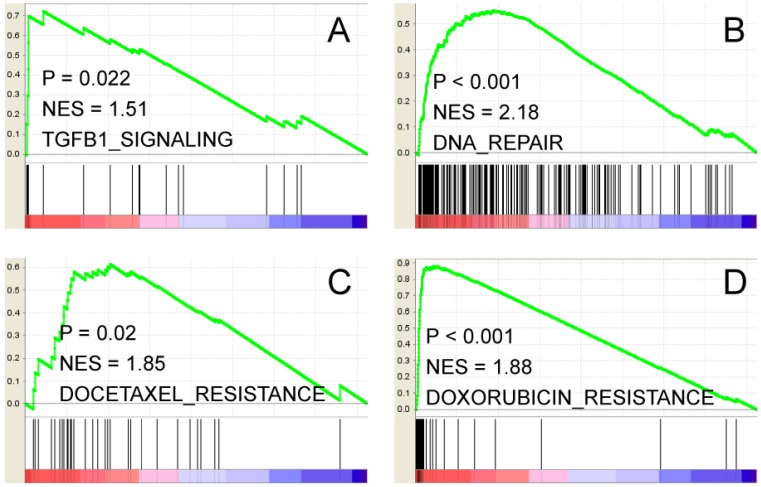
Two class GSEA indicates that TGF-beta pathway (A), DNA repair (B), Docetaxel and Doxorubicin resistance (C, D) gene signatures are enriched in genes overexpressed in FOXM1+/PLAU+ subgroup. NES stands for normalized enrichment score.

Furthermore, two class Gene Set Enrichment Analysis (GSEA) results indicated that the above differentially expressed genes are enriched in several oncogenic signaling pathways. For instance, TGF-beta signal (Figure 3A, NES = 1.51, P=0.022); DNA repair signature (Figure 3B, NES = 2.18, p<0.001); Docetaxel and Doxorubicin resistance signatures (Figure 3C and D, NES=1.85, p=0.02; NES=1.88, p<0.001, respectively) are significantly enriched. Thus, FOXM1 and PLAU may function coordinately to promote gastric cancer progression and therapeutic resistance through multiple signaling pathways.

**Figure 4:**
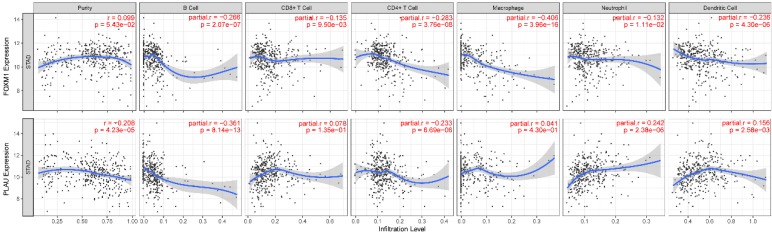
Association between FOXM1, PLAU and immune cell infiltration. Correlation r or purity adjusted r value and p value are presented in the figure. The blue line is the fitting curve.

We also show that FOXM1 is negatively correlated with infiltration of all six types of immune cells while PLAU is negatively correlated with B cells and CD4+ T cells; positively correlated with neutrophil and dendritic cells (Figure 4). Gene network analysis showed that FOXM1 was involved in RAS signaling pathway while PLAU was associated with blood coagulation, wound healing and cell-matrix adhesion (Supplementary Figure 6).

### Potential therapeutic options for FOXM1+PLAU+ patient group

As mentioned, FOXM1 and PLAU are indicators of poor prognosis with shorter OS and RFS time in the gastric cancer, and results from microarray also indicated FOXM1+PLAU+ related genes are enriched in TGF-beta, DNA repair, MAPK and drug resistance signaling pathways. To search for the potential therapeutic drugs or chemicals targeting FOXM1+PLAU+ related molecular patterns, LINCs dataset was explored. As is shown in Supplementary Figure 7 and Supplementary Table 6, CDK inhibitors, mTOR inhibitors, JAK2 inhibitors, PI3K inhibitor, IKK inhibitor and IGF-1R inhibitors etc. could potentially reversed the gene expression patterns of FOXM1+PLAU+ or FOXM1-PLAU- groups. As a conclusion, the predicted inhibitors which could reverse the gene expression pattern in the different FOXM1/PLAU status groups may serve as potential therapeutic options in the gastric cancer in the future.

## Discussion

Gastric cancer remains the third cause of mortality in the worldwide^1^. Because of the high recurrence rate and poor prognosis of GC, there still one half of the patients suffer surgery but with no changes in long term survival^55^. Therefore, more efforts should be encouraged to develop individualized therapies. Previously, we found that overexpression of FOXM1 and PLAU were associated with gastric cancer progression and poor prognosis. In the current study, several bioinformatics approaches are employed to explore the molecular mechanisms underlying FOXM1+/PLAU+ related malignant phenotype and potential therapeutic options.

We show that FOXM1 and PLAU are overexpressed in 17 cancer types including GC and the expression of these two genes are positively correlated with each other. Kaplan-Meier curve analysis using pooled gastric cancer dataset indicate that FOXM1+PLAU+ subgroup predict the worst prognosis, while FOXM1-PLAU- subgroup have the best OS and RFS. Genes related with PLAU+FOXM1+ subgroup play crucial roles in various signaling pathways, including cell cycle, DNA repair pathway, TGF-beta pathway and chromosome remodeling. FOXM1 is negatively correlated with infiltration of all six types of immune cells while PLAU is negatively correlated with B cells and CD4+ T cells; positively correlated with neutrophil and dendritic cells. Gene network analysis showed that FOXM1 was involved in RAS signaling pathway while PLAU was associated with blood coagulation, wound healing and cell-matrix adhesion. Moreover, literature mining indicates that FOXM1 could upregulated PLAUR expression^12^. If PLAU and FOXM1 were co-overexpressed, then the PLAU-PLAUR pathway would be over-activated. These could account for PLAU+FOXM1+ associated poor prognosis of GC patients. Furthermore, genomic and proteomic differences between FOXM1+PLAU+ and FOXM1-PLAU- groups were also computed using TCGA GC data. Several mutations and proteins of key signaling pathways are enriched or overexpressed in FOXM1+PLAU+ group.

Besides the well-known signaling pathways that FOXM1 and PLAU involved in, our results also indicate that FOXM1 and PLAU may participate in the JAK-STAT3, DNA repair and drug resistance (Docetaxel and Doxorubicin) in GC. JAK2-STAT3 axis activation is the feature in many solid tumor and play an important role in cell proliferation and microenvironment changed^56^, ^57^. Drugs targeting on JAK2-STAT3 pathway in autoimmune disease have been shown in phase 3 trial, but no response was seen in solid tumor even in the phase 1 trial^58^, ^59^. Dysfunction of DNA repair pathway cause the accumulating DNA damage, and induce the tumorigenesis. And therefore more effort has been make on the anticancer drugs which focused on the DNA repair^60^-^62^.

Subsequently, we analyzed the potential inhibitors that could potentially reverse the gene expression patterns of the FOXM1+/PLAU+ subgroup and FOXM1-/PLAU- subgroup. As shown in Figure 3, inhibitors targeting cell cycle (CDK), PI3K-AKT, NF-κB, JAK2, TGF and mTOR would be effective as suggested by LINCs. Among these inhibitors, BMS-754807 is a reversible and potent inhibitor of insulin-like growth factor 1 receptor (IGF-1) and *in vivo* experiments show that combination with BMS-754807 and cetuximab improve the outcome of BMS-754807 single treatment^63^. TPCA-1 was reported as direct inhibitor of STAT3 and NF-κB, and is effective in a subgroup of NSCLC^64^. The potential therapy capacity of FOXM1 and PLAU is not limited in the gastric cancer, also suitable for the NSCLC.

Drug resistance is an important problem influence the prognosis of cancer. Suppression of FOXM1 and PLAU could sensitize the pancreatic cancer cell death by inducing DNA damage^65^. Our results also indicated that the genes related to the docetaxel or doxorubicin resistance were overexpressed in FOXM1+/PLAU+ subgroup. Therefore, those predicted inhibitors which could reverse the gene expression patterns of the FOXM1+PLAU+ subgroup may worth exploring as new therapeutic options for gastric cancer.

In summary, PLAU+FOXM1+ could serve as effective prognostic biomarkers and potential therapeutic targets for GC. Due to the additive effect of these two genes, screening for drugs or chemicals that targeting the expression patterns PLAU+FOXM1+ subgroup may exert important clinical impact on GC management. Nevertheless, this study is an in-silico study using multiple computational and bioinformatics approaches, wet lab experiments should performed to further confirm our findings.

## Supplementary Material

Supplementary figures

Supplementary tables.
