# Supplementary material for: FOXM1 functions collaboratively with PLAU to promote gastric cancer progression
Source: J Cancer. 2020 Jan 1;11(4):788–94. doi: 10.7150/jca.37323 (PMC6959008; doi:10.7150/jca.37323)
Supplement: Supplementary file 1 — Supplementary figures [file jcav11p0788s1.pdf]

Supplementary Materials

Supplementary Figure 1. Box plot of FOXM1 and PLAU expression in 32 cancer types. FOXM1 and PLAU are overexpressed in gastric cancer (marked in yellow box). ***: p<0.001, **: p<0.01, *: p<0.05. Red represents expression value in cancer while blue represents expression in normal control. Purple represents SKCM metastases group.

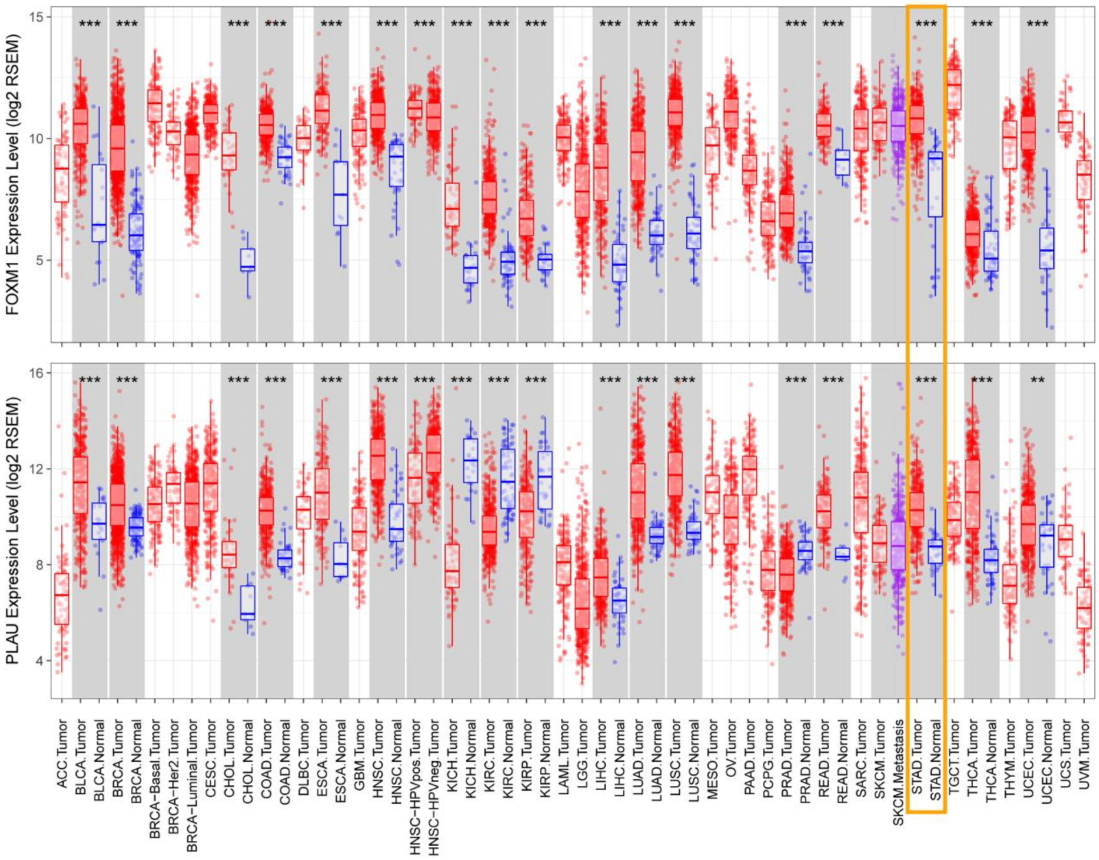

Supplementary Figure 2. FOXM1 and PLAU are overexpressed in gastric (A, data from GSE27342, red represents cancer, blue represents normal control); FOXM1 and PLAU expression are negatively correlated with methylation level, Pearson r and p values are presented (B); FOXM1 and PLAU expression and copy number variation, FOXM1 expression is associated with CNV (C); Correlation of FOXM1 and PLAU expression adjusted by tumor cell proportion (D). ***: p<0.001, **: p<0.01, *: p<0.05.

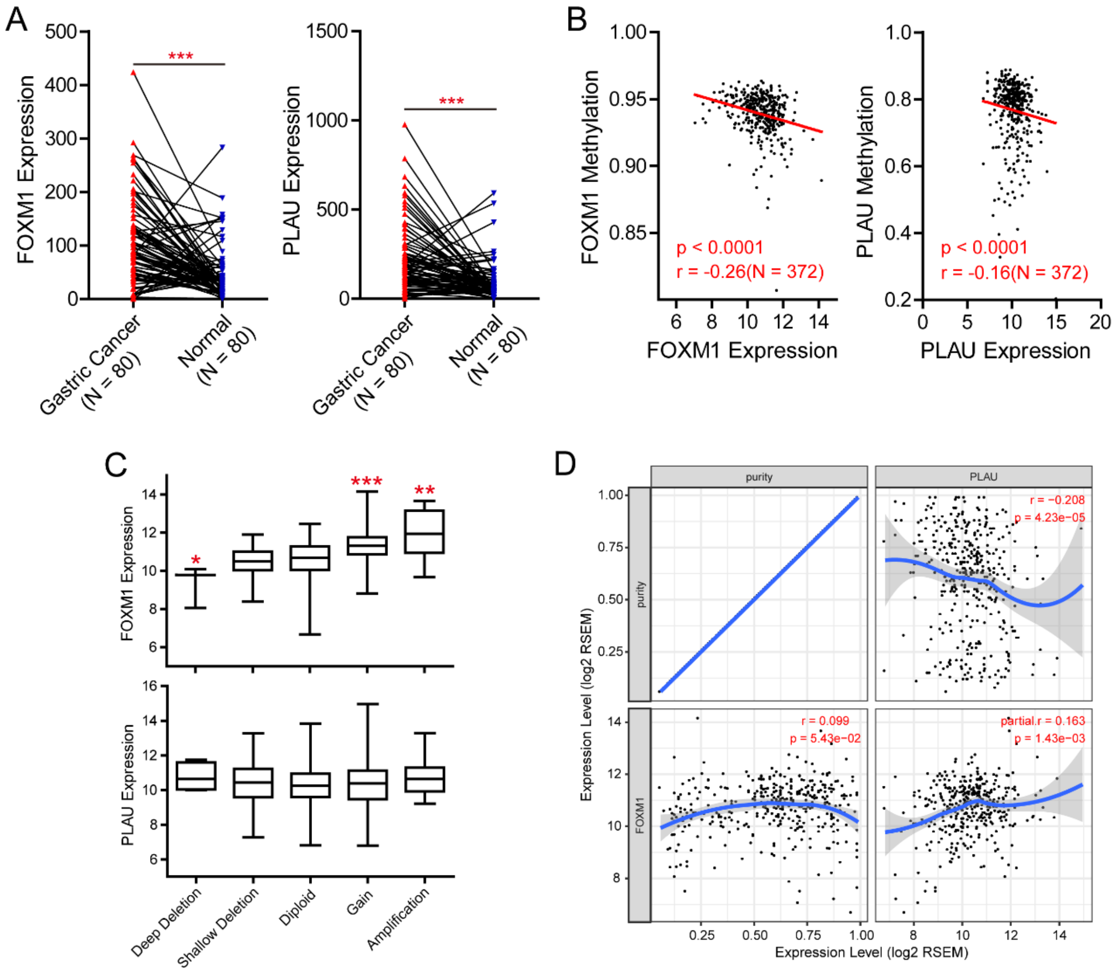

Supplementary Figure 3. The expression of FOXM1 and PLAU are not associated with tumor stages, P = 0.826 and 0.0785, respectively.

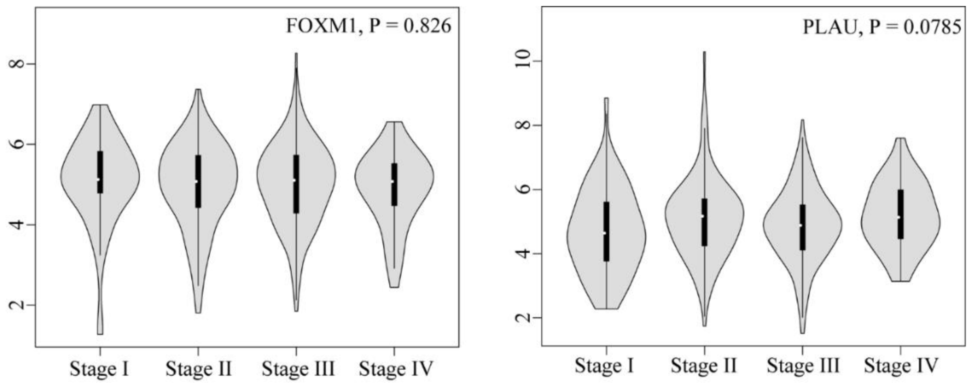

Supplementary Figure 4. The mutation count in FOXM1+PLAU+ group is significantly higher than FOXM1-PLAU- group (A). JARID2, ZFHX4 and SYNE1 mutations are significantly enriched in FOXM1+PLAU+ group (B).

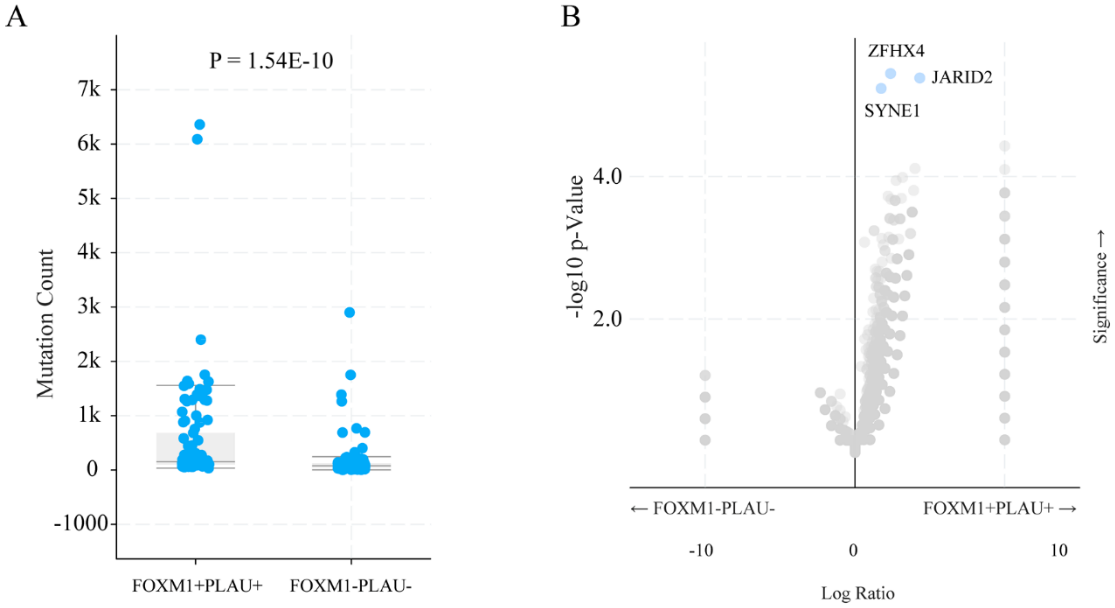

Supplementary Figure 5. Mutation frequency and CNA frequency comparison between FOXM1+PLAU+ and FOXM1-PLAU- groups. As is shown in this graph, blue represents FOXM1+PLAU+ group and red represents FOXM1-PLAU- group.

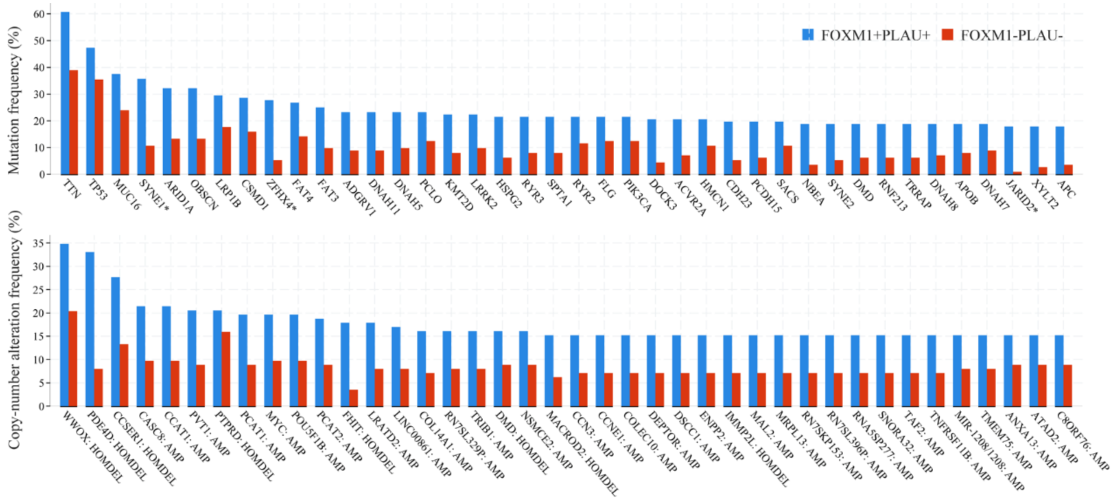

Supplementary Figure 6. Gene interaction network and function enrichment of FOXM1 and PLAU. Different line colors represent different signaling pathways.

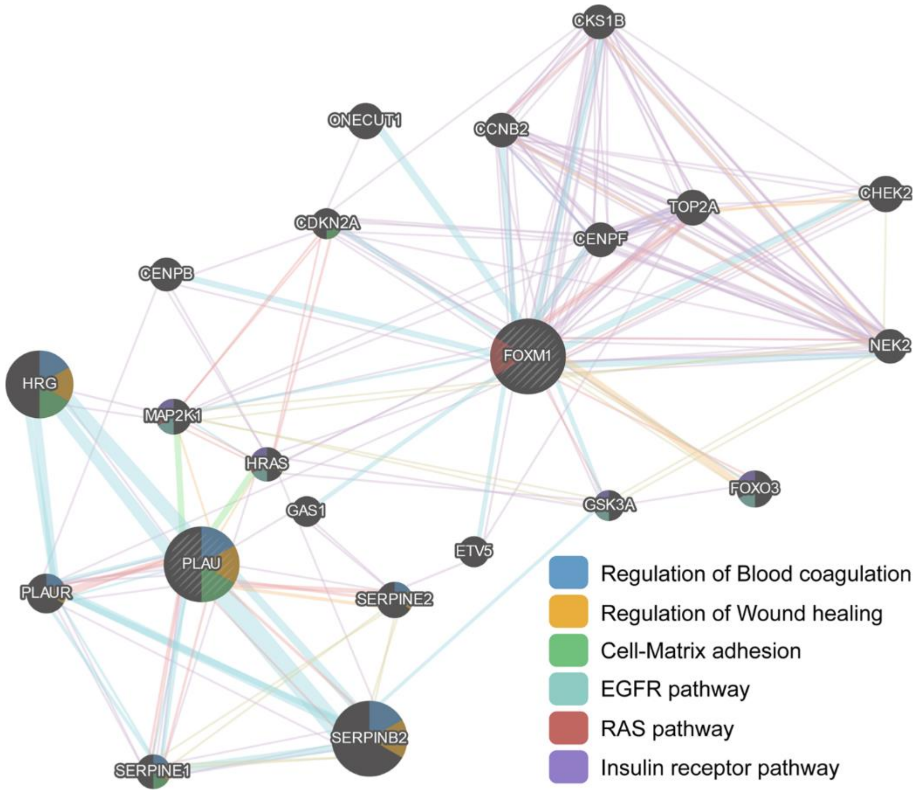

Supplementary Figure 7. Data mining results from LINCs dataset suggests several inhibitors that could reverse the expression pattern of genes differentially expressed in FOXM1+/PLAU+ subgroup compared with FOXM1-/PLAU- subgroup.

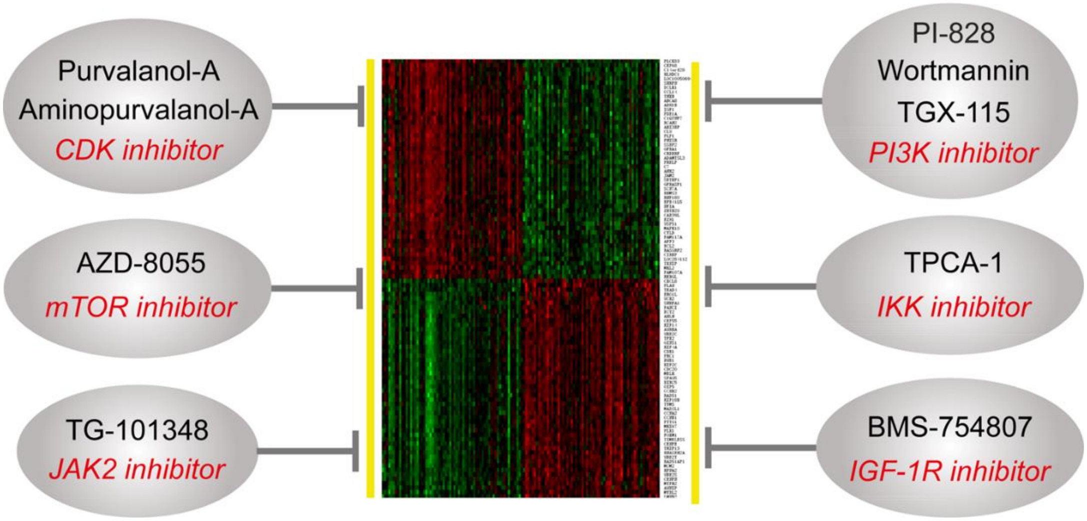

Supplementary Tables

Supplementary Table 2. Top 50 up/down genes in FOXM1+PLAU+ group compared with FOXM1-PLAU- group.

Supplementary Table 1. Number of patients in each group in figure 1.

Supplementary Table 3. The differences of Mutation and clinical attributes between FOXM1+PLAU+ and FOXM-PLAU- groups.

Supplementary Table 4. Mutations enriched in FOXM1+PLAU+ and FOXM1+PLAU+ group.

Supplementary Table 5. Protein changes of key signaling pathways from RPPA data of TCGA.

Supplementary Table 6. Drugs targeting genes expression patterns of FOXM1+PLAU+ group.
