# Supplementary material for: FOXM1 functions collaboratively with PLAU to promote gastric cancer progression
Source: J Cancer. 2020 Jan 1;11(4):788–94. doi: 10.7150/jca.37323 (PMC6959008; doi:10.7150/jca.37323)
Supplement: Supplementary file 2 — Supplementary tables. [file jcav11p0788s2.zip › Supplementary Table 2.docx]

Supplementary Table 2. Top 50 up/down genes in FOXM1+PLAU+ group compared with FOXM1-PLAU- group

| Gene Symbol | Gene Description | Up/Down |
| --- | --- | --- |
| FOXM1 | forkhead box M1 | Up |
| KPNA2 | karyopherin alpha 2 | Up |
| PRC1 | protein regulator of cytokinesis 1 | Up |
| SPAG5 | sperm associated antigen 5 | Up |
| UBE2S | ubiquitin-conjugating enzyme E2S | Up |
| PLAU | plasminogen activator, urokinase | Up |
| AURKA | aurora kinase A | Up |
| TIMELESS | timeless circadian clock | Up |
| KIF14 | kinesin family member 14 | Up |
| CDK1 | cyclin-dependent kinase 1 | Up |
| UBE2C | ubiquitin-conjugating enzyme E2C | Up |
| TRIP13 | thyroid hormone receptor interactor 13 | Up |
| SNRPA1 | small nuclear ribonucleoprotein polypeptide A | Up |
| TPX2 | TPX2, microtubule-associated | Up |
| BUB1 | BUB1 mitotic checkpoint serine/threonine kinase | Up |
| RAD51AP1 | RAD51 associated protein 1 | Up |
| MCM2 | minichromosome maintenance complex component 2 | Up |
| MELK | maternal embryonic leucine zipper kinase | Up |
| CXCL8 | chemokine (C-X-C motif) ligand 8 | Up |
| KIF2C | kinesin family member 2C | Up |
| MAD2L1 | MAD2 mitotic arrest deficient-like 1 (yeast) | Up |
| KIF4A | kinesin family member 4A | Up |
| UBE2T | ubiquitin-conjugating enzyme E2T (putative) | Up |
| MTFR2 | mitochondrial fission regulator 2 | Up |
| ERO1L | ERO1-like (S. cerevisiae) | Up |
| FANCI | Fanconi anemia, complementation group I | Up |
| ECT2 | epithelial cell transforming 2 | Up |
| CENPE | centromere protein E, 312kDa | Up |
| CCNA2 | cyclin A2 | Up |
| CCNB1 | cyclin B1 | Up |
| CEP55 | centrosomal protein 55kDa | Up |
| ANLN | anillin, actin binding protein | Up |
| CENPN | centromere protein N | Up |
| MYBL2 | v-myb avian myeloblastosis viral oncogene homolog-like 2 | Up |
| PTTG1 | pituitary tumor-transforming 1 | Up |
| AUNIP | aurora kinase A and ninein interacting protein | Up |
| MKI67 | marker of proliferation Ki-67 | Up |
| CDC20 | cell division cycle 20 | Up |
| TYMS | thymidylate synthetase | Up |
| PLK1 | polo-like kinase 1 | Up |
| OIP5 | Opa interacting protein 5 | Up |
| GINS1 | GINS complex subunit 1 (Psf1 homolog) | Up |
| TEAD4 | TEA domain family member 4 | Up |
| CCNB2 | cyclin B2 | Up |
| BIRC5 | baculoviral IAP repeat containing 5 | Up |
| KIF18B | kinesin family member 18B | Up |
| LMNB2 | lamin B2 | Up |
| RAD51 | RAD51 recombinase | Up |
| UCK2 | uridine-cytidine kinase 2 | Up |
| RNASEH2A | ribonuclease H2, subunit A | Up |
| NFIA | nuclear factor I/A | Down |
| GFRA1 | GDNF family receptor alpha 1 | Down |
| C14orf28 | chromosome 14 open reading frame 28 | Down |
| TNXB | tenascin XB | Down |
| ADH1B | alcohol dehydrogenase 1B (class I), beta polypeptide | Down |
| RERGL | RERG/RAS-like | Down |
| ANK2 | ankyrin 2, neuronal | Down |
| MAPK10 | mitogen-activated protein kinase 10 | Down |
| SNRPN | small nuclear ribonucleoprotein polypeptide N | Down |
| PNISR | PNN-interacting serine/arginine-rich protein | Down |
| SCN7A | sodium channel, voltage-gated, type VII, alpha subunit | Down |
| CREBRF | CREB3 regulatory factor | Down |
| MKL2 | MKL/myocardin-like 2 | Down |
| FAM107A | family with sequence similarity 107, member A | Down |
| PLCXD3 | phosphatidylinositol-specific phospholipase C, X domain containing 3 | Down |
| BCL2 | B-cell CLL/lymphoma 2 | Down |
| ADAMTSL3 | ADAMTS-like 3 | Down |
| AFF3 | AF4/FMR2 family, member 3 | Down |
| CCL14 | CCL15-CCL14 readthrough (NMD candidate) | Down |
| RBMS3 | RNA binding motif, single stranded interacting protein 3 | Down |
| C1QTNF7 | C1q and tumor necrosis factor related protein 7 | Down |
| LOC284112 | uncharacterized LOC284112 | Down |
| CYLD | cylindromatosis (turban tumor syndrome) | Down |
| SSBP2 | single-stranded DNA binding protein 2 | Down |
| PLP1 | proteolipid protein 1 | Down |
| C7 | complement component 7 | Down |
| KLHDC1 | kelch domain containing 1 | Down |
| ZBTB20 | zinc finger and BTB domain containing 20 | Down |
| JAM2 | junctional adhesion molecule 2 | Down |
| LOC100506948 | uncharacterized LOC100506948 | Down |
| SETBP1 | SET binding protein 1 | Down |
| USP51 | ubiquitin specific peptidase 51 | Down |
| RCAN2 | regulator of calcineurin 2 | Down |
| CEP68 | centrosomal protein 68kDa | Down |
| PDE1A | phosphodiesterase 1A, calmodulin-dependent | Down |
| EZH1 | enhancer of zeste 1 polycomb repressive complex 2 subunit | Down |
| PRELP | proline/arginine-rich end leucine-rich repeat protein | Down |
| RASGRP2 | RAS guanyl releasing protein 2 | Down |
| GPRASP1 | G protein-coupled receptor associated sorting protein 1 | Down |
| ABI3BP | ABI family, member 3 (NESH) binding protein | Down |
| CLU | clusterin | Down |
| RNF180 | ring finger protein 180 | Down |
| DCLK1 | doublecortin-like kinase 1 | Down |
| TXNIP | thioredoxin interacting protein | Down |
| CIRBP | cold inducible RNA binding protein | Down |
| EPB41L5 | erythrocyte membrane protein band 4.1 like 5 | Down |
| FAM117A | family with sequence similarity 117, member A | Down |
| ABCA8 | ATP-binding cassette, sub-family A (ABC1), member 8 | Down |
| CAB39L | calcium binding protein 39-like | Down |
| IGF1 | insulin-like growth factor 1 (somatomedin C) | Down |
