# Supplementary material for: FOXM1 functions collaboratively with PLAU to promote gastric cancer progression
Source: J Cancer. 2020 Jan 1;11(4):788–94. doi: 10.7150/jca.37323 (PMC6959008; doi:10.7150/jca.37323)
Supplement: Supplementary file 2 — Supplementary tables. [file jcav11p0788s2.zip › Supplementary Table 6.docx]

Supplementary Table 6. Drugs targeting genes expression patterns of FOXM1+PLAU+ group

| Drugs | Function | CMAP score |
| --- | --- | --- |
| AZD-8055 | mTOR inhibitor | 99.791 |
| PI-828 | PI3k inhibitor | 99.778 |
| TG-101348 | JAK2 inhibitor | 99.681 |
| BMS-754807 | IGF-1R inhibitor | 99.637 |
| TGX-115 | PI3k inhibitor | 99.913 |
| Aminopurvalanol-A | CDK inhibitor | 99.27 |
| TPCA-1 | IKK inhibitor | 99.238 |
| Wortmannin | PI3K inhibitor | 99.646 |
| Purvalanol-A | CDK inhibitor | 98.276 |
